# The “Depressive” Attributional Style Is Not That Depressive for Buddhists

**DOI:** 10.3389/fpsyg.2017.01003

**Published:** 2017-06-28

**Authors:** Michelle T. Liu, Fei Wang, Kaiping Peng

**Affiliations:** ^1^Department of Psychology, School of Social Science, Tsinghua UniversityBeijing, China; ^2^Department of Biomedical Engineering, School of Medicine, Tsinghua UniversityBeijing, China

**Keywords:** depressive attributional style, Buddhist, karma, ultimate internal controllability, psychological adjustments

## Abstract

Numerous studies have shown that depression-prone people are characterized by a chronic style of attributing failures to internal, stable, and global causes, sometimes labeled as the “depressive attributional style.” Much less is known, however, about how social-cultural factors such as religious beliefs might modulate these processes. In the current study, we hypothesized that Buddhism’s view of ultimate internal controllability plays a buffering role against the depressive attributional style and reduces its negative impacts. We administrated measures of attributional styles and psychological adjustments to a sample of Chinese Buddhists as well as a control group recruited in China. Data analyses showed that Buddhists were more likely to attribute bad outcomes to internal, stable, and global causes, but their well-being was less affected by it. Thus, these results indicate that the “depressive” attributional style is not that depressive for Buddhists, after all.

## Introduction

### Attributional Styles

Forty years ago, Seligman proposed the learnt helplessness model of depression, which proposed that control over the environment is a fundamental need for any organism, and if one is repeatedly exposed to unavoidable painful stimuli, one will come to expect that such events are uncontrollable and develop hopelessness and depression as a result (Hiroto and Seligman, 1975). This model was later reformulated to the Attributional Style theory (Abramson et al., 1978), which identified three dimensions of attribution of positive and negative outcomes: (a) internality: whether the outcome is due to internal (oneself) or external causes (others or circumstances); (b) stability: whether the outcome is due to stable or temporary causes; (c) globality: whether the outcome is due to global (generalizable to different situations) or specific (limited to the current situation) causes. A chronic style of attributing failures to internal, stable, and global causes, sometimes labeled as the ‘depressive attributional style’ is characteristic of depression-prone people (Seligman, 2002).

Initial empirical support for these theoretical propositions has been mixed (Coyne and Gotlib, 1983). For example, Zuroff (1981) found that while depressed participants made more internal attributions for failure than non-depressed participants, in absolute terms, they still favored external over internal attributions for failure. Many of the negative findings, however, might be attributed to inadequate statistical power (Robins, 1988). Meta-analyses did show that the depressive attributional style is a reliable predictor of depression and other indices of well-being (Sweeney et al., 1986; Gladstone and Kaslow, 1995; Joiner and Wagner, 1995).

### Influences of Cultural Factors on Attributional Styles

One less-investigated aspect of the depressive attributional style, however, is how socio-cultural factors, such as religious beliefs, may moderate its impact on well-being. The majority of studies on this topic were conducted in a Western cultural context, while Easterners, under the influences of religious belief systems such as Buddhism, may behave differently in this regard.

Empirically, cross-cultural research has shown that there is both similarity and variability across cultures in the attributional process. A meta-analysis of 266 studies (Mezulis et al., 2004) showed that, compared with Westerners, Easterners display a smaller self-serving attributional bias (making more internal, stable, and global attributions for positive events than negative events). However, the maladaptive nature of the depressive attributional style seems to be generalized across cultures. For example, while Chinese tend to take more responsibility for interpersonal failures and less credit for interpersonal success than Americans, the relationship between attributional style and depression are similar across both samples (Anderson, 1999).

While these studies shed important insights into how social-cultural factors may influence the attributional processes, it is unknown to what extent these cross-cultural patterns originate from different worldviews or belief systems. In the current study, we focus on Buddhism, which is one of the most influential Eastern religious systems, and examine how Buddhist beliefs affect attributional styles and their impacts on well-being. In the next section, we briefly review aspects of Buddhist beliefs that are relevant to attributional processes.

### A Theoretical Analysis of Buddhist Beliefs in Relation to Attributional Styles

One central aspect of Buddhist beliefs is a meritocracy-based retribution system, commonly referred to as “karma.” In this system, there is a successive cycling between life and death, and intentional actions in this life lead to consequences in the next life. More specifically, it is believed that behaviors are decided by thoughts and each thought is one or a combination of 51 kinds of mental factors (Skt. caitasika), and these mental factors belong to one of three categories: ethical, unethical, and neutral (Vasubandhu, *Treatise of Thirty Verses of Consciousness-Only, Skt. Triṃśikāvijñaptikārikāḥ*). As a result, every thought and behavior also possesses ethical, unethical, or neutral attributes. Ethical (wholesome) actions lead to pleasant/enjoyable life experiences, unethical (unwholesome) actions lead to unpleasant/suffering life experiences, and neutral actions lead to neither pleasant nor unpleasant life experiences (in future reincarnations; and for some schools of Buddhism, also in this life).

Consider, for example, the action of using a knife. For a surgeon, using a knife is ethical and leads to the retribution of pleasant experiences such as good health. In contrast, a robber’s action of using a knife is unethical and leads to unpleasant experiences such as a sense of fear and deprivation. Using a knife to cut fruits, on the other hand, is neutral, and the retribution is neither pleasant nor unpleasant.

The consequence of the karma system is a totally internal, global, and generalized attributional style. Everything has causes and supportive conditions, and there is a robust link between a person’s intentional actions and future consequences. However, it should be noted that, in many cases, the “person” is a process rather than a single identity – a person in this life is the result of an action undertaken by another person in another life, both of whom are not identical.

Based on the literature of the depressive attributional style, one may predict that such a style of attribution would negatively affect Buddhists’ mental health. However, we argue here that another core idea of Buddhist beliefs, that is, the view of “no-self,” would lead to ultimate internal controllability, which could buffer against such an attributional style and reduce its negative impacts. Based on non-duality of the “perceived” and “perceiver,” Buddhism views controllability as not objective but perceived. The uniqueness of the Buddhist worldview is “no self” (Skt. anātman), in which the “self” (ātman) has a very clear definition: (a) it is a whole unit, undividable into smaller components; (b) permanent, does not change over time; (c) dominant over its own existence, not being subject to other conditions. Because we cannot find anything that possesses these characteristics of “having the characteristics of an own nature (Skt. svabhāva),” all phenomena are thus of the nature of “no-self,” commonly referred to as “emptiness” (Skt. śūnyatā). No phenomenon exists rigidly but is in a constant process of arising, changing, and ceasing, including us as a “person” (Skt. pudgala).

However, the initiative of Buddhism was not phenomenology. It did not aim to explain “how the universe came to be” but “why we experience dissatisfaction with life.” It proposes the famous Four Noble Truths (Muller, 2017):

(1) The truth of suffering (Skt. Duḥka): suffering is the lot of the six states of existence;
(2) The truth of the arising of suffering (Skt. Samudaya): suffering is aggregated (or exacerbated) by afflicted mental states;
(3) The truth of the cessation of suffering (Skt. Nirodha): cessation (Nirvāṇa) is attainable as the extinction of desire and its consequences and the leaving of the sufferings of mortality as void and extinct;
(4) The truth of the path to the cessation of suffering (Skt. Marga): there’s a path to the cessation of suffering, i.e., the Eightfold Correct Path.

The core spirit of the Four Noble Truths is the ultimate internal controllability over the relief of all dissatisfaction with life. The underlying power of control is not by holding on to the existence of a “self,” but the opposite, i.e., dissolving the notion of “real existence” in a “perceiver.” The concept of “no self” introduces the interdependence of all things, and equipped with the “right view” and “ethical behaviors,” one is a major influencer of his/her own fate.

One implication of the ultimate internal controllability is that, in the face of hardship in life, Buddhists would take a more rational and flexible/dialectic approach to an understanding of the situation and the formation of a constructive solution. For example, when facing a failed financial investment, a Buddhist might attribute the failure to several factors. Firstly, the main cause would be his own (past) mentality of greed and correspondent actions leading to material scarcity. Secondly, greed and other unethical mental factors, such as delusion, worked together to drive him to make the wrong investment decision. Thirdly, Buddhist wisdom emphasizes the cultivation of insight, which enables one to immediately identify supportive conditions to make things happen, and avoid unsupportive environments or timing. Such would be the reflection of the failed financial investment by a Buddhist. Furthermore, when dealing with negative emotions that arise during a hardship, a Buddhist might try to analyze the underlying mental factors and then deal with them with counteractive mental factors and other behavioral interventions. In the example of “financial loss due to greedy decisions or lack of insight,” a Buddhist would emphasize on practicing sharing/altruistic actions such as donation; endurance instead of being in denial of the situation, and diligently work on gaining the knowledge necessary to avoid future irrational mistakes, the stability of one’s mind so that it does not get easily disturbed, and finally and most importantly, the nature of emptiness, from which all things including fortune, manifest. The above mental factors include *aspiration* (to gain wealth as resources for truth seeking and to benefit others), *concentration*, *discernment* (to be able to choose according to the mechanism of causality), *resolve* (thorough understanding of the tenets), *anti-greed*, and *anti-delusion*. All of the above are exemplary positive character traits acquired through learning and practice. In addition to all the ethics, the backbone of such practices is a logic/rationale that has been tested and endorsed by heated debates over thousands of years within sects and with non-Buddhists. Karma is both a logical conclusion and an empirical, gradual path of practice.

Therefore, even though Buddhists possess an extremely stable, internal, and global attributional style, which fits right into the so-called “depressive” attributional style, their view of ultimate internal controllability might produce quite a different output than learned helplessness, thereby buffering against the risks of depression and other mental problems.

### Related Empirical Works and the Current Study

The above section reviewed two aspects of Buddhist beliefs that are relevant to attributional styles. Based on this analysis, we could predict that the so-called “depressive” attributional style is not that depressive for Buddhists. To the best of our knowledge, there has been no empirical study directly testing this hypothesis. Three lines of relevant literature, however, may shed some insights into the issue. First, research has shown that belief in karma is associated with poorer physical and mental health of survivors of trauma experience (Davidson et al., 2005; Levy et al., 2009). For example, Davidson et al. (2005) surveyed a representative United States community sample of 1969 respondents and found that 5% of them strongly agreed on the belief in karma and reincarnation. Critically, belief in karma was associated with more extensive traumatization, such as abuse, rape, and loss of a family member through violent death, as well as more severe posttraumatic stress symptoms. Levy et al. (2009) found that among Sri Lankan tsunami survivors (most of whom were lay Buddhists), belief in karma and a depressive attributional style were independently associated with poor health. At first glance, these results seem to be contradictory to our hypothesis. However, karmic belief is only one of the core components of the Buddhist worldview and, as we have proposed above, the idea of ‘no-self’ and the resulting ultimate internal controllability are also critical. Endorsement of these core ideas requires a deep understanding and sufficient theoretical knowledge of Buddhism, which might be beyond the scope of the lay beliefs held by the participants in these studies. Also, these studies did not directly test the interaction between Buddhist beliefs and the depressive attributional style, which is our central hypothesis. Furthermore, these studies focused on a special subclinical/clinical population. Second, in recent decades, there has been a rise of interest in adopting Buddhist concepts and practices, such as detachment, mindfulness, and meditation, into psychotherapy, which resulted in techniques such as Mindfulness-based Stress Reduction (Kabat-Zinn, 1990), Dialectical Behavior Therapy (Shearin and Linehan, 1994), and Mediation Awareness Training (Van Gordon et al., 2014). These techniques have been shown to be effective in the treatment of conditions such as depression and borderline personality disorder and in the enhancement of psychological health (Linehan et al., 2006; Keng et al., 2011; Strauss et al., 2014; Van Gordon et al., 2016), which may be considered as indirect evidence of Buddhist worldviews’ buffering effect against mental problems. Third, research has shown that self-forgiveness, especially its ‘true’ form (i.e., acknowledging one’s wrongdoing and accepting responsibility), is associated with better psychological adjustment (Tangney et al., 2005). Intriguingly, Tangney et al. (2005) found little difference in the level of self-forgiveness across various religious groups, which included Buddhists. The generalizability of this result, however, is limited by the fact that their sample consisted of only college students and their friends or parents.

In the current study, we aim to directly test the relation between attributional styles and indices of psychological adjustment in a sample of Buddhists as well as a control group. If Buddhist beliefs do play a buffering role against the detrimental effect of the depressive attributional style, we would expect a significant moderation effect of Buddhist beliefs on the relationship between the depressive attributional style and psychological well-being, with a reduced or absent association between the two variables in Buddhists (vs. the control group).

## Materials and Methods

### Participants

All the participants were ethnically Chinese, and the survey and recruitment were conducted in Chinese. One-hundred thirty-seven Buddhists were recruited through social connections and social media, including lay Buddhists, monastics, and academic professionals with a Buddhism-related profession. Among them, 133 resided in Mainland China, 2 resided in Hong Kong, 1 resided in Taiwan, and 1 resided in the United States. In addition to the subject’s self-claim as a Buddhist by faith, a screening questionnaire was designed to measure the fundamental ideas of a Buddhist worldview as well as the schools/sects the subject subscribed to, and the root literature of the identified schools/sects, as a cross-validation of one’s self claim (Appendix A). One was included in the Buddhist group only if he/she satisfied the following standards: (1) one should believe in reincarnation; (2) one should agree with the following statement: “All sufferings in life are due to one’s ignorance of the real causal mechanism regarding how things work; thus erroneous opinions, words, and behaviors are produced.”; (3) a Māhayāna Buddhist should believe that one can understand the nature (ontology) of the universe – that is “no-self” or emptiness (empty of its own being) – whereas a Theravadin might not, which is consistent with the self-reporting of school/sects because they simply should not care; (4) a Yogācāra Buddhist would agree with “External things do not exist. They are but the work of our mind, like a painting to the painter” – that is the view of Vasubandhu, the founder of Yogācāra – whereas a Māhayāna Buddhist might or might not agree, the reason being that they think the mind is also empty of self-existence, which is also in line with Nāgārjuna’s view that any implication of self-existence must be denied, and is something a Theravadin would not care about, the consistency of which we can check with regard to their self-reported school and root literature; (5) one should identify some representative literature as his/her root literature (i.e., it best depicts his/her version of a Buddhist world view). If one reported a school he/she identified him/herself with, the root literature and the school should be consistent with common Buddhist school classification standards. This screening test ensured that the Buddhist sample endorsed the core tenets. Seventeen of the participants failed to pass this test, leaving 120 participants in the Buddhist group (117 resided in Mainland China, 2 resided in Hong Kong, and 1 resided in Taiwan). One-hundred seventy-seven non-Buddhists, who were all ethnically Chinese and resided in Mainland China, were recruited through social media as the control group. We asked them about their experience with Buddhism to ensure that none of them endorsed Buddhist worldviews. Both groups of the participants took part in the study voluntarily without compensation.

**Table 1** presents the demographics of the two groups.

Statistical analyses showed that the two groups significantly differed in these demographics (for gender: χ^2^ = 4.94, *p* = 0.03; for age: *t* = 8.16, *p* < 0.001; for educational level: χ^2^ = 24.79, *p* < 0.001). Therefore, we controlled these variables as covariates in the subsequent analyses.

**Table 1:** Demographics of the Buddhist and control groups.

	Buddhists (*n* = 120)	Control (*n* = 177)
Gender	50 male, 70 female	97 male, 80 female
Age	39.54 ± 9.93 years	30.68 ± 8.64 years
Educational level		
Middle school or below	5 (4.23%)	2 (1.13%)
High school	13 (10.83%)	6 (3.39%)
University/College	66 (55.00%)	144 (81.36%)
Graduate or above	36 (30.00%)	25 (14.12%)

### Measures

#### All Measures Were Administered in Chinese

##### Attributional Style Questionnaire (ASQ)

The ASQ (Peterson et al., 1982), which measures one’s attributional styles for hypothetical good and bad outcomes, is the most widely used self-reported measure of depressive attributional style. In the current study, we employed the Chinese version of the ASQ (Huang, 2007), which contains six positive and six negative events. After reading each of the events, participants completed three items measuring the three dimensions of internality, stability, and globality. The three dimension scores were computed by summing up the six items for each of the dimensions, separately for bad and good outcomes. Next, a composite score was computed by summing up the three dimension scores. A higher score on any of these measures indicates that one is more inclined toward the depressive attributional style. For the Buddhist group, the alpha coefficients of the three dimensions were 0.73, 0.68, and 0.81 for good outcomes; and 0.74, 0.75, and 0.87 for bad outcomes. For the control group, the alpha coefficients of the three dimensions were 0.62, 0.56, and 0.76 for good outcomes; and 0.57, 0.62, and 0.74 for bad outcomes. The alpha coefficients of the total scale of good outcomes were 0.86 and 0.83, respectively, for the Buddhist and the control groups. The alpha coefficients of the total scale of bad outcomes were 0.89 and 0.83, respectively, for the Buddhist and the control groups.

##### Beck Depression Inventory (BDI)

The BDI (Beck et al., 1961) is a self-reported measure of depression. In the current study, we employed the Chinese version of the BDI (Zhang et al., 1990). Participants read 21 questions and choose one of four answers, which increase in intensity. The alpha coefficient of the BDI was 0.82 and 0.89 for the Buddhist and control groups, respectively.

##### Positive and Negative Affect Scale (PANAS)

The PANAS is a self-reported measure of affective well-being (Watson and Clark, 1999). In the current study, we employed the Chinese version of the PANAS (Qiu, 2006). Participants read 10 positive and 10 negative affect words and report how often they recently experienced these affects using a 5-point scale from *1 = Never* to *5 = Always*. For the Buddhist group, the alpha coefficients were 0.87 and 0.83 for positive and negative affects, respectively. For the control group, the alpha coefficients were 0.88 and 0.87 for positive and negative affects, respectively.

##### Satisfaction with Life Scale (SWLS)

The SWLS (Diener et al., 1985) is a self-reported measure of the cognitive evaluation of one’s life. In the current study, we employed the Chinese version of the SWLS (Wu, 2007). Participants read five statements and rate their extent of agreement with a 7-point Likert-like scale from *1 = Totally disagree* to 7 = *Totally agree*. The alpha coefficient of the SWLS was 0.86 and 0.87 for the Buddhist and control groups, respectively.

### Procedure

Participants completed the measures through a commercial online survey platform. Informed consent was obtained from all participants prior to completion of the online survey.

## Results

### Group Differences in Attributional Styles

**Table 2** summarizes the scores of attributional styles of the two groups. A series of general linear modeling analyses were performed to assess group differences, with gender, age, and three dummy variables representing educational level as covariates. For bad outcomes, Buddhists were more likely to attribute them to internal, stable, and global causes and scored higher on the composite score (*F*s > 8.13, *p* < 0.005). For good outcomes, the group differences in the dimension scores and the composite score were not significant (*F*s < 0.36, *p*s > 0.55).

**Table 2:** Comparisons between the Buddhist and control groups on attributional styles.

	Buddhists	Control	*F*
**Attributions for bad outcomes**			
Internal	31.91 ± 7.03	28.43 ± 5.41	22.87^∗∗^
Stable	26.78 ± 7.70	23.40 ± 6.26	14.42^∗∗^
Global	26.53 ± 10.23	24.26 ± 8.10	8.13^∗∗^
Composite	85.21 ± 19.95	76.08 ± 16.53	20.19^∗∗^
**Attributions for good outcomes**			
Internal	31.07 ± 7.27	31.42 ± 5.24	0.06
Stable	32.27 ± 7.40	33.01 ± 5.51	0.22
Global	30.37 ± 8.56	31.73 ± 6.23	0.36
Composite	93.70 ± 20.69	96.17 ± 14.98	0.26

*^∗∗^p < .001.*

### Group Differences in Psychological Adjustment

**Table 3** summarizes the scores of the indicators of psychological adjustment of the two groups. General linear modeling analyses (controlling for gender, age, and three dummy variables representing educational level) showed that Buddhists reported higher life satisfaction with their lives and less symptoms of depression, but had less positive affect and more negative affect (*F*s > 4.04, *p*s < 0.05).

**Table 3:** Comparisons between the Buddhist and control groups on psychological adjustment.

	Buddhists	Control	*F*
Depression	8.32 ± 6.31	10.10 ± 8.37	4.04^∗^
Positive affect	32.30 ± 7.24	35.27 ± 6.81	8.11^∗∗^
Negative affect	24.13 ± 6.30	21.97 ± 7.08	9.65^∗∗^
Life satisfaction	21.70 ± 6.74	17.80 ± 6.33	7.49^∗∗^

*^∗^p < 0.05, ^∗∗^p < 0.01.*

### Moderating Effects of Buddhist Beliefs on the Relations between Attributional Styles and Psychological Adjustment

To examine how the relation between the depressive attributional style and psychological adjustment might differ between the two groups, we performed a series of moderation analyses using the PROCESS program (Hayes, 2013). In each of the analyses, the dependent variable was one of the indicators of psychological adjustment, and the predictors were the composite ASQ score for bad outcomes and participant group, as well as their interaction term. Gender, age, and three dummy variables of educational level were included into the model as covariates. Bootstrap simulations were performed 5000 times to determine the bias-corrected confidence level of the effects.

**FIGURE 1:**
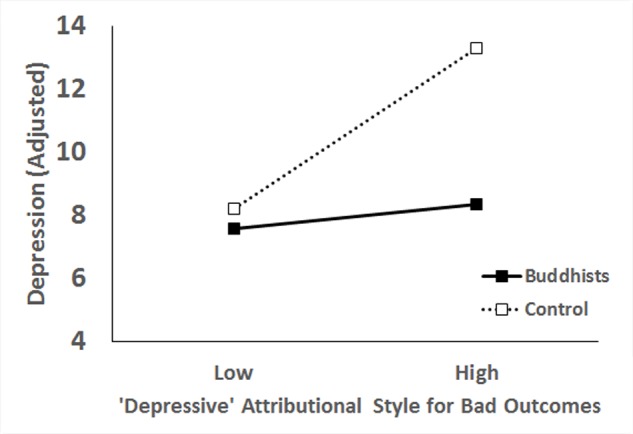
Interaction effect of the depressive attributional style and Buddhist beliefs on depression.

For depression, the main effect of the depressive attributional style was significant, *B* = 0.09, *t* = 3.71, *p* < 0.001, 95% *CI* [0.04–0.14]; which was qualified by a significant interaction effect, *B* = 0.12, *t* = 2.45, *p* = 0.01, 95% *CI* [0.02–0.21]. Simple main effect analysis showed that for the control group the depressive attributional style was a significant predictor of depression, *B* = 0.14, *t* = 4.08, *p* < 0.001, 95% *CI* [0.07–0.20]; but the effect was not significant in the Buddhist group, *B* = 0.02, *t* = 0.60, *p* = 0.55, 95% *CI* [-0.05–0.09] (**Figure 1**).

**FIGURE 2:**
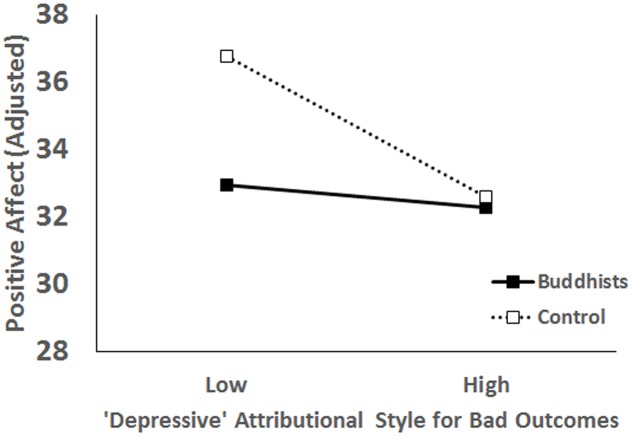
Interaction effect of the depressive attributional style and Buddhist beliefs on positive affect.

For positive affect, the main effect of the depressive attributional style was significant, *B* = -0.07, *t* = -3.29, *p* < 0.001, 95% *CI* [-0.12 to -0.03]; which was qualified by a significant interaction effect, *B* = -0.09, *t* = -2.12, *p* = 0.04, 95% *CI* [-0.18 to -0.01]. Simple main effect analysis showed that for the control group, the depressive attributional style was a significant predictor of positive affect, *B* = -0.11, *t* = -3.59, *p* < 0.001, 95% *CI* [-0.17 to -0.05]; but the effect was not significant in the Buddhist group, *B* = -0.02, *t* = -0.57, *p* = 0.57, 95% *CI* [-0.08–0.04] (**Figure 2**).

**FIGURE 3:**
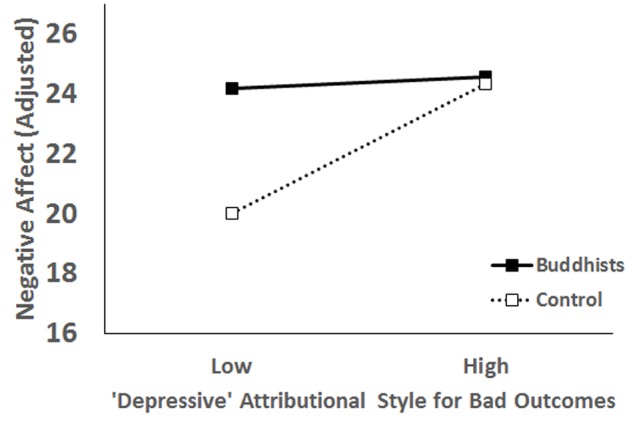
Interaction effect of the depressive attributional style and Buddhist beliefs on negative affect.

For negative affect, the main effect of the depressive attributional style was significant, *B* = 0.07, *t* = 3.45, *p* < 0.001, 95% *CI* [0.03–0.12]; which was qualified by a significant interaction effect, *B* = 0.11, *t* = 2.53, *p* = 0.01, 95% *CI* [0.02–0.18]. Simple main effect analysis showed that for the control group, the depressive attributional style was a significant predictor of negative affect, *B* = 0.12, *t* = 3.94, *p* < 0.001, 95% *CI* [0.06–0.18]; but the effect was not significant in the Buddhist group, *B* = 0.01, *t* = 0.35, *p* = 0.73, 95% *CI* [-0.05–0.07] (**Figure 3**).

For life satisfaction, the main effect of the depressive attributional style was not significant, *B* = -0.02, *t* = -0.92, *p* = 0.36, 95% *CI* [-0.06–0.02]. The interaction effect was also not significant, *B* = 0.01, *t* = 0.33, *p* = 0.75, 95% *CI* [-0.07–0.10].

## Discussion

By administrating the ASQ in a sample of Buddhists and a control group, we found that Buddhists were more likely to attribute bad outcomes to internal, stable, and global causes. This finding is in line with the concept of “karma” – the meritocracy-based retribution system that is central to Buddhist beliefs. In the karma system, everything has causes, pleasant or unpleasant, and life is directly related to wholesome or unwholesome behavior. Under the guidance of these beliefs, Buddhists are less likely to show the self-serving bias – the common tendency to attribute successes to internal factors and attribute failures to external factors (Larson, 1977), and are more willing to take responsibility for negative consequences.

In the previous literature, such a “depressive” attributional style has constantly been shown to be associated with depression and decreased well-being (Sweeney et al., 1986; Gladstone and Kaslow, 1995; Joiner and Wagner, 1995; Seligman, 2002). Based on these studies, one might predict that Buddhists would have lowered levels of psychological adjustment than the control group. Our data suggests otherwise; while reporting less positive affect and more negative affect, they had more favorable evaluations of their own lives and showed fewer symptoms of depression. Overall, the Buddhist and the control groups manifested psychological adjustment of comparable levels.

Most critically, Buddhist beliefs significantly moderated the attributional style’s impact on three indicators of psychological adjustment. While the depressive attributional style was associated with a higher level of depression, less positive affect, and more negative affect in the control group, these associations were absent in the Buddhist group. This buffering effect of Buddhist beliefs might be attributed to its view of ultimate internal controllability. Buddhism’s non-duality of the “perceived” and “perceiver” leads to a unique view of control, i.e., to obtain the power of control, the real existence of the perceiver has to be dissolved. By following the Eightfold Path, one can ultimately decide his/her own fate and realize enlightenment. Equipped with such views, even though Buddhists tends to attribute bad outcomes to internal, stable, and global causes, they will not be caught in the paralyzing grip of pessimism, but try to reach a rational understanding of the situation and resolve it.

One unexpected result is that life satisfaction was not associated with attributional style in both groups. This is in contrast with a previous study showing that the depressive attributional style leads to lowered life evaluations (e.g., Chang and Sanna, 2007). However, in this study the magnitude of attributional style’s correlation with life satisfaction was indeed lower than its correlations with other indicators of well-being. We speculate that such patterns might be due to the construct of life satisfaction reflecting the cognitive aspect of well-being (Diener et al., 2003), whereas the depressive attributional style is more closely related to the affective aspect of well-being. More research is needed to replicate this finding and further explore its underlying mechanism.

Overall, the current study shows that the well-documented link between the internal, stable, and global styles of attribution and psychological adjustment is not universal; at least for Buddhists, the depressive attributional style is not that depressive. Although the concept of a “depressive” attributional style was initially proposed as a general underlying mechanism of depression, it was mostly based on studies conducted in Western cultures. Buddhism, while also stressing the responsibility of one’s actions, seems to alter the attributional processes’ implications for personal well-being. On a broader level, the current study provides a rare example of how religious worldviews may affect psychological processes in a cultural context, which has often been overlooked in previous cross-cultural studies (Tarakeshwar et al., 2003). Future studies could try to expand the research of attributional styles and other psychological processes into different religious groups and explore other socio-cultural factors as boundary conditions.

It should be noted that the current study has some limitations. First, due to the scope of the paper, we described the Buddhist ideology system in a rather simplified way. In fact, there are many different schools of Buddhism, whose teachings and practices vary in many aspects. Although we believe that the core ideas relevant to our research question are similar across these schools, future studies may empirically examine how ideological differences of various Buddhist schools modulate the attributional process. Second, our selection of the Buddhist sample was mainly based on their acceptance of Buddhist doctrines, while in most main schools practice is at least as important as theoretical knowledge. Given previous research on Buddhism-related psychotherapy techniques (Keng et al., 2011; Van Gordon et al., 2014), it is possible that the buffering role of Buddhist beliefs against the depressive attributional style is partially due to the practice of meditation or other forms of mindful reflexivity. Future research may try to differentiate the relative contributions of Buddhist teachings and practices. Third, for the control group, we only asked about their experience with Buddhism to make sure that they were not Buddhism believers. It is possible that some of them endorsed worldviews from other religions (e.g., Christianity), which might also affect the attributional process. Nevertheless, the depressive attributional style’s deleterious effect on psychological adjustment was successfully replicated in this group. Forth, a few subscales of the ASQ had lower alpha coefficients (<0.60). This might partially be due to the small number of items (6) for each subscale. Nonetheless, our main analyses focused on the composite scores, which had adequate internal reliabilities (>0.80) for both groups. Fifth, the current study relied on self-reported measures. Although such measures are direct and intuitive, people might behave differently when facing negative outcomes in real life. Future research could try to conceptually replicate the current findings in experimental settings. For example, one might engage Buddhists and non-Buddhists with certain self-relevant tasks (e.g., intelligence tests; Heider et al., 1958) and provide them with either positive or negative feedback to observe how they react differently. Based on the theoretical analyses of Buddhist beliefs and the current findings, we might predict that when facing negative feedback, Buddhists would be more likely to make internal, stable, and global attributions, but at the same time put more effort to improve their performances. Another potentially fruitful avenue is to look further into the mechanism of Buddhist attributional processes and identify pathways that mediate its buffering effect against the depressive attributional style.

## Ethics Statement

The study has been approved by the ethics committee of Department of Psychology, School of Social Science, Tsinghua University. Informed consent was obtained from all participants prior to completion of the online survey. No vulnerable populations were involved.

## Author Contributions

ML developed the research idea with KP and FW. ML performed the data collection. ML and FW performed data analyses and wrote the first draft. All authors contributed to the revision of the manuscript.

## Conflict of Interest Statement

The authors declare that the research was conducted in the absence of any commercial or financial relationships that could be construed as a potential conflict of interest.
