# Supplementary material for: The “Depressive” Attributional Style Is Not That Depressive for Buddhists
Source: Front Psychol. 2017 Jun 28;8:1003. doi: 10.3389/fpsyg.2017.01003 (PMC5487417; doi:10.3389/fpsyg.2017.01003)
Supplement: Supplementary file 1 [file Table_1.DOCX]

***Supplementary Material***

**Burn Serum Increases** ***Staphylococcus aureus* Biofilm Formation via Oxidative Stress**

**Supeng Yin^1^, Bei Jiang^1^, Guangtao Huang^1^, Yali Gong^1^, Bo You^1^, Zichen Yang^1^, Yu Chen^1^, Jing Chen^1^, Zhiqiang Yuan^1^, Ming Li^2^, Fuquan Hu^2^, Yan Zhao^2*^ and Yizhi Peng^1*^**

***Correspondence:** Yizhi Peng. E-mail: [yizhipen@sina.com](mailto:yizhipen@sina.com)**,** Yan Zhao. E-mail: hnyanyanxp@aliyun.com

**Supplementary Table 1**

**Supplementary Table 1.** **Primers used for quantitative real-time PCR**

| **Primers** | **Sequence (5’-3’)** |
| --- | --- |
| 16S-F | GCGCTGCATTAGCTAGTTGGT |
| 16S-R | TGGCCGATCACCCTCTCA |
| *fnbA*-F | CCAGGTGGTGGTCAGGTTAC |
| *fnbA*-R | TGTGCTTGACCATGCTCTTC |
| *fnbB*-F | ACCTGCTAAAGAAGAACC |
| *fnbB*-R | CGTAATAACGCTAAACCTA |
| *clfA*-F | TTTCAACAACGCAAGATA |
| *clfA*-R | GCTACTGCCGCTAAACTA |
| *clfB*-F | TTTGGGATAGGCAATCATCA |
| *clfB*-R | TCATTTGTTGAAGCTGGCTC |
| *icaA*-F | TACTATTTCGGGTGTCTTCA |
| *icaA*-R | CAAAGACCTCCCAATGTT |
| *icaR*-F | CCAAATTTTTGCGAAAAGGA |
| *icaR*-R | TACGCCTGAGGAATTTTCTG |
| *rbf*-F | ACGCGTTGCCAAGATGGCATAGTCTT |
| *rbf*-R | AGCCTAATTCCGCAAACCAATCGCTA |
| *sarA*-F | GCACAACAACGTAAAAAAATCGAA |
| *sarA*-R | TTCGTTGTTTGCTTCAGTGATTC |
| *saeS*-F | AATCCAGAACCACCCGTTTT |
| *saeS*-R | ACGCCACTTGAGCGTATTTT |
| *rot*-F | TCGCTTTCAATCTCGCTGAA |
| *rot*-R | CGACACTGTATTTGGAATTTTGCA |
| *sigB*-F | CCTACTGTAATCGGTGAAATC |
| *sigB*-R | GTCCCATTTCCATTGCTTC |
| *agrC*-F | ACCCGATGAAGTAAGTAGCA |
| *agrC*-R | TAGACCTAAACCACGACCTT |
| *cidA*-F | AATTTCGGAAGCAACATCCA |
| *cidA*-R | CTTCCCTTAGCCGGCAGTAT |
| *atl*-F | TTTGGTTTCCAGAGCCAGAC |
| *atl*-R | TTGGGTTAAAGAAGGCGATG |
| *sodA*-F | CCAATGTAGTCAGGGCGTTT |
| *sodA*-R | GTTCAGGTTGGGCTTGGTTA |
| *sodM*-F | CGTAATAATGGCGGTGGT |
| *sodM*-R | TCTTGGTTTGGCGTTGTC |
| *katA*-F | TGCTGCAAGGGCGTTTAT |
| *katA*-R | TCAGTTGGGAATGGTGGC |
| *ahpC*-F | GCATGACCATTCAGATGCAA |
| *ahpC*-R | CCAATTCCGTCAGCGTTAAT |
